# Single-blind, placebo controlled randomised clinical study of chitosan for body weight reduction

**DOI:** 10.1186/s12937-016-0122-8

**Published:** 2016-01-08

**Authors:** VR Trivedi, MC Satia, A. Deschamps, V. Maquet, RB Shah, PH Zinzuwadia, JV Trivedi

**Affiliations:** 1 Ethicare Clinical Trial Services, Titanium City Centre, 100 Feet Road, Ahmedabad, 380015 Ahmedabad, India; 2 KITOZYME, Parc Industriel des Hauts-Sart, Zone 2, Rue de Milmort 680, 4040 Herstal, Belgium; 3 Poojan Multispecialty Hospital, Gurukul Road, Memnagar, Ahmedabad, 380052 India; 4 DHL Research Centre, Nr. Shivranjani Cross Roads, Satellite, Ahmedabad, 380015 India; 5 SAL Hospital, Drive-in Road, Ahmedabad, 380054 India

**Keywords:** Chitosan, Obesity, Randomised, Body weight, Anthropometric measurements, Body composition, HbA1C

## Abstract

**Background:**

Chitosan is a dietary fibre which acts by reducing fat absorption and thus used as a means for controlling weight. Weight loss clinical trial outcomes, however, have contradictory results regarding its efficacy. The primary objective of the present study was to evaluate the efficacy and safety of a chitosan from fungal origin in treatment of excess weight in the absence of dietary restrictions.

**Methods:**

A phase IV, randomised, multicentre, single-blind, placebo-controlled, clinical study was conducted by administering chitosan capsules (500 mg, five/day) and indistinguishable placebo capsules as daily supplements to 96 overweight and obese subjects for 90 days. The study participants were divided in 2:1 ratio to receive either chitosan (*n* = 64) or placebo (*n* = 32). Efficacy was assessed by measuring body weight, body composition parameters, anthropometric measurements, HbA1C level and lipid profile at day 45 and day 90. Also, short form-36 quality of life (QoL) questionnaire was assessed to evaluate improvement in life-style and dietary habits were recorded for calorie intake. Safety was assessed by evaluating safety parameters and monitoring adverse events.

**Results:**

The mean changes in body weight were -1.78 ± 1.37 kg and -3.10 ± 1.95 kg at day 45 and day 90 respectively in chitosan group which were significantly different (*p* < 0.0001) as compared to placebo. BMI was decreased by10.91 fold compared to placebo after 90 day administration. In concert with this, there was also reduction in body composition and anthropometric parameters together with improvement in QoL score. Chitosan was also able to reduce HbA1C levels (below 6 %) in subjects who had initial higher values. The mean caloric intake shows that there was no change in dietary habits of subjects in both groups. Lipid levels were unaffected and all adverse events were mild in nature and unrelated to study treatment.

**Conclusion:**

Chitosan from fungal origin was able to reduce the mean body weight up to 3 kg during the 90 day study period. Together with this, there was also improvement in body composition, anthropometric parameters and HbA1C, reflecting overall benefits for the overweight individuals. Additionally, there was also improvement in QoL score. It was safe and well tolerated by all subjects.

**Trial registration:**

CTRI/2014/08/004901.

## Background

Obesity is a prevalent health hazard in developed and developing countries and is closely associated with various pathological disorders, including diabetes, hypertension, and cardiovascular diseases [1]. In simple terms, it may be defined as a state of imbalance between calories ingested versus calories expended which would lead to excessive or abnormal fat accumulation [2]. Newer classifications of obesity are based on simple measures such as waist hip ratio, total adiposity and intra-abdominal fatness. Anthropometric indexes such as the body mass index (BMI) and waist-to-hip ratio (WHR) remain the most commonly used tools for assessing body composition because of their simplicity and low cost [3].

International Association for the Study of Obesity (IASO), and the International Obesity Task Force (IOTF) estimated, in a study jointly conducted in 2010, that approximately 1.5 billion adults were overweight with around 475 million obese adults. Globally, IASO/IOTF also estimated that up to 10 % (~200 million) school aged children were either overweight or obese, 20 % of which are in European Union [4].

Treatment of obesity includes lifestyle-based intervention (diet, exercise, and behaviour therapy) and medical or surgical intervention (pharmacotherapy or bariatric surgery). There are several approaches through which pharmacotherapies are directed to treat obesity. These include, limiting the absorption of food, suppressing appetite and reducing food intake, and altering metabolism or increasing energy expenditure [5]. However, their use is controversial as these pharmacotherapies are known to have significant adverse effects [6, 7] and a consensus on the optimal clinical use of these pharmacological agents is not fully established yet, and additional large clinical studies are needed [8]. One of the most efficient and safe method would be a reduction in fat intake, as obesity is directly associated with total fat consumption [9]. One such substance which acts by reducing the dietary absorption of fat is chitosan.

Chitosan is a popular dietary fibre often used to prevent dietary fat absorption as a means for controlling weight. It is a cationic polysaccharide produced by deacetylation (hydrolysis of the N-acetyl-D-glucosamine units) of the biopolymer chitin, which is derived from the cuticles of crustaceans such as shrimp, crab, and lobster [10], or from the cell wall of mushroom [11]. The properties of chitosan are similar to cellulose [12]. The cationicity of chitosan is due to the non-acetylated amines of the polyglucosamine residues that make up the polymer chains [13]. Due to its cationic nature, chitosan binds to negatively charged lipids, hence reducing their gastrointestinal uptake and also potentially lowering serum cholesterol [14]. There are number of reports which demonstrate that chitosan binds dietary lipids and bile acids in in-vitro, pre-clinical and human studies [15–19]. The effects of chitosan as a treatment for overweight and obesity has been evaluated in many clinical trials of great variability in terms of study design and quality resulting to somewhere inconsistent results [20–25]. However, most of the randomised double-blind placebo-controlled trials have reported that it may decrease body weight and serum lipids [10, 20–23, 25, 26] while a few studies have found no effect of chitosan on clinical outcomes [12, 24]. Among the reasons for these variable outcomes are inadequate dosage of chitosan and/or variation of caloric intake throughout the study (changes in food habits/inconsistent diet). In order to investigate the effect of chitosan at a daily dose of 2.5 g, we conducted a randomised, placebo-controlled clinical trial to evaluate the safety and efficacy of KiOnutrime-CsG® capsules (Chitosan, 500 mg) in treatment of excess weight in the absence of dietary modifications. KiOnutrime-CsG® (KitoZyme, Belgium) is a non-animal chitosan obtained from the cell walls of the non-genetically modified *Aspergillus niger* mycelium, a by-product of citric acid production. KiOnutrime-CsG® is an alternative to crustacean-derived chitosan.

## Methods

This was a 90 days, phase IV, randomised, multicentre, single-blind, placebo-controlled, clinical study conducted at four hospital sites in cities of Ahmedabad and Bangalore in India. The study protocol and protocol-related documents were approved by the institutional ethics committee of each site before initiating any trial related activity.

### Study participants

**Fig. 1:**
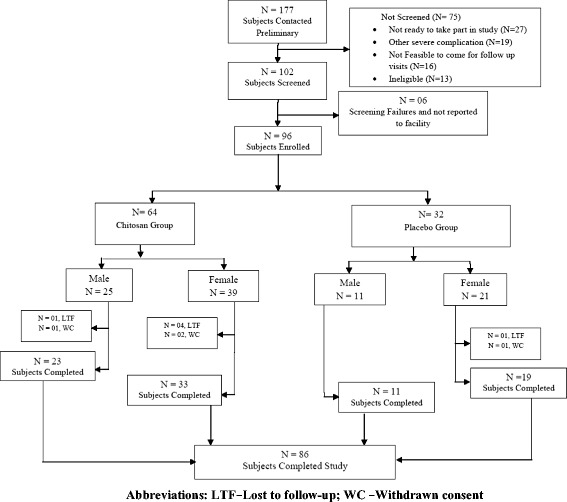
Disposition of subjects

For preliminary phase screening, a total of 177 study participants either included in hospital’s databases or under doctor’s referral were initially contacted. Out of this, 102 subjects were selected for screening and 75 subjects were not selected based on the reasons described in Fig. 1. Subjects of both genders were screened based on inclusion criteria and were included in the trial if they were aged 18 to 65 years, who expressed interest to participate in the study, had BMI between 26 and 35 (both inclusive), willing to comply with the study schedule and procedure. The exclusion criteria were history of surgical procedure(s) in the past 6 months, history of invasive fat reduction procedure (e.g., liposuction, abdominoplasty, mesotherapy) within the past one year, history of subcutaneous injections (e.g., heparin, insulin) within the past one month, subject on stable dose of metformin, pioglitazone or on glucagon-like peptide analogues, subjects with history of bleeding disorder, subjects with history of allergy to chitosan, subjects with treatment of fat soluble vitamins and minerals or other dietary aids, subjects with poorly controlled diabetes mellitus or hypertension, subjects having inflammatory diseases of the gastrointestinal tract, female subjects who are pregnant or willing to get pregnant, not ready to use contraceptive measures during the trial period, or is breast feeding or in preparations used in the treatment of menopause disorders and subjects who are taking or has taken diet pills or supplements within the past 30 days. All study participants provided voluntary written informed consent before initiating the screening procedures. Audio-video recording of the entire informed consent process was carried out according to the schedule Y of Indian GCP.Out of the 102 screened subjects, 96 subjects were enrolled in the study. Each enrolled subjects received Subject Information Sheet and Subject diary containing details of food consumption and drug compliance. They were instructed not to change their routine dietary habits.

### Randomisation, medication dosing and dispensing

Study subjects were randomized in a 2:1 ratio to receive chitosan (KiOnutrime-CsG® capsules) or indistinguishable placebo capsules. This 2:1 ratio was decided to limit the exposure of placebo formulation to half of the subjects only. The study medications were dispensed in pre-labelled identical bottles to all sites according to individual site randomisation schedule generated using a computerised random number generator with mixed block sizes to prevent the identity of treatment assignment. There was no stratification by sex or other demographic variables.The randomisation codes were kept in an individually sealed, opaque envelope and broken only after the completion of data lock procedures.

The chitosan used in the study was chitosan derived from *Aspergillus niger*. Each capsule of KiOnutrime-CsG® contained 500 mg chitosan with excipients (magnesium stearate and colloidal silicone dioxide). In case of placebo, chitosan was replaced with 500 mg microcrystalline cellulose powder and the excipients were colloidal silicone dioxide, colour yellow oxide ofiron and colour natural caramel (in order to match KiOnutrime-CsG® colour). Each bottle of study medication contained 75 capsules for total 15 days administration. Subjects were instructed to take one capsule in the morning, 2 capsules 15 min before lunch and 2 capsules 15 min before dinner with a glass of water. They were instructed to fill up the time of drug administration and the number of capsules taken in the subject diary. Subjects were also instructed to visit the study centre every 15 days to receive new medication containers and to assess medication compliance from the subject diary. Dosage compliance was assessed counting the unused quantity of medication from returned bottles. Medication compliance was considered reached when there was 90 % capsules intake over a period of 90 days capsules administration. Subjects were also advised to maintain their normal routine diet and to record the type and amount of food consumed (for periods of 5 days between days 1–5, days 41–45 and days 86–90) in the food diary provided, to calculate and analyse the daily caloric value.

### Study visits and evaluations

Subjects visited the study centre four times for evaluation of study parameters: during screening (Visit 1; Day -5 to 0), enrolment/randomisation visit (Visit 2; Day 1), follow-up visit (Visit 3; Day 45 ± 2) and end of study visit (Visit 4; Day 90 ± 3). At each study visit except randomisation visit, demographic data, anthropometric determinations (includes upper abdominal circumference, hip circumference, waist circumference and waist to hip ratio), body composition (BMI, body fat, visceral fat, muscle mass), HbA1c, lipid parameters (triglyceride, HDL, LDL, VLDL), and biochemistry data (urea, serum creatinine, SGPT, SGOT) were evaluated to determine safety and efficacy of KiOnutrime-CsG®. Physical examinations and vital signs (radial pulse, blood pressure, respiratory rate, and body temperature) were carried out at all visits.

Body weight and body composition parameters were measured using calibrated Body Fat Monitor (Tanita Corporation, Japan; Model BC 601), which assesses body composition indirectly by multifrequency bioelectrical impedance analysis. Anthropometric determinations were made using non-stretch measuring tape to the nearest 0.1 cm. Fasting blood was collected onsite and then transferred within 30 min to central pathology laboratory (Dr Lal Pathlabs at Ahmedabad and Bangalore, India). Serum samples were separated (4000 rpm, 15 min, 4 °C), immediately frozen and stored at -20 ^°^C until analysed. This was thawed and allowed to come to room temperature just before the estimation of HbA1c by immunoturbidimetry assay (Bio-Rad Laboratories, India; Model D-10), lipid parameters by direct enzymatic colorimetric assay and biochemistry determinations (SGPT and SGOT by IFCC method; urea by urease kinetic method; creatinine by buffered Jaffe’s method) (Roche Diagnostic Limited, Switzerland; Model Cobas Integra 400 plus). All analyses were completed within 12 h of blood collection and all methods were validated by three freeze-thaw cycles.

Study participants also completed a SF-36 (Short Form 36) health-related quality of life (QoL) questionnaire [27, 28] at each visit except the randomisation visit. As part of the questionnaire, SF-36 assessed subjects’ health-related quality of life. The questionnaire consisted of eight multi-item dimensions. They were physical functioning (PF), limitations due to physical problems (Role-Physical), vitality (VT), bodily pain (BP), social functioning (SF), limitations due to emotional problems (Role-Emotional), mental health (MH), and general health (GH). The scores from these dimensions were further grouped into physical and mental components expressed as Physical Component Summary (PCS) and Mental Component Summary (MCS) scores. The PCS score reflected physical morbidity and adaptation to disease, whereas the MCS score referred to mental morbidity and adaptation.

Assessment of daily average calorie intake was also carried out by recording the subjects’ diet from their food diary to confirm whether there wasany influence of diet on the observed weight loss. This was assessed by an independent dietician. The average calorie intake for the period of day 1–5, day 41–45 and day 86–90 was calculated for both the groups.

### End points and measures of outcomes

The primary efficacy end point was reduction in body weight in kilograms on day 45 and day 90 compared to baseline. Secondary outcome measures include mean changes in body composition data (BMI, body fat, visceral fat, muscle mass), anthropometric values (change in upper abdominal circumference, hip circumference, waist circumference and waist to hip ratio), lipid profile and HbA1c levels.

Safety was evaluated by clinically and physically observing and reporting adverse events (AE) and assessing changes in vital signs, and biochemistry parameters. Change in SF-36 QoL scale from baseline was also assessed to evaluate safety and efficacy of KiOnutrime-CsG® capsules.

### Statistical analyses

The sample size of this study was based on the primary objective of reduction in the body weight after 90 days treatment with chitosan. Based on a power of 95 % due to higher variation among Indian subjects and a type I error rate of 0.05 (2-tailed), a sample size of at least 72 subjects (48 in KiOnutrime-CsG® group and 24 in placebo group after considering randomization ratio of 2:1) is required to detect a clinically significant difference of 2 kg reduction in body weight after chitosan treatment with a standard deviation of 2.69 based on previous published study [1]. Considering dropout rate of 20 %, adjusted sample size will be 90 (60 subjects in KiOnutrime-CsG® group and 30 subjects in placebo group after considering randomization ratio of 2:1).

All the statistical analyses were carried out using SAS v9.0 (SAS Institute Inc, Cary, NC, USA). Results were expressed as mean ± standard deviation (SD). The distribution of the variables was investigated using the Kolmogorov-Smirnov test. To evaluate the effect of chitosan capsules on the investigated variables, *P* value for between groups comparison was calculated using unpaired t- test or Mann Whitney test based on the distribution of data. In case of within group comparison, data were analysed using paired *t*-test or Wilcoxon test depending upon the distribution of data. *P* values of less than 0.05 were considered as statistically significant difference between and within treatment groups.

## Results

### Disposition of subjects

Of the 102 subjects screened, a total of 96 subjects were enrolled in the study. Of these, 64 subjects were randomly assigned to the chitosan group and 32 to the placebo group. Five subjects from chitosan group and one from placebo group were lost to follow-up; while three subjects from chitosan group and one from placebo group withdrew their consent during the course of the study. A total of 86 subjects completed the study. Figure 1 describes the disposition of the study subjects.

### Demography& subject characteristics

Baseline demographic characteristics (mean ± SD) for subjects in the chitosan group were, age 35.53 (± 11.23) years; weight and height 80.13 (± 11.47) kg and 1.61 (± 0.10) m, respectively and for the placebo group were, age 36.28 (± 10.49) years; weight and height 80.54 (± 12.68) kg and 1.61 (± 0.09) m,respectively. There was no significant difference between the baseline demographics of study participants in each treatment group (Table 1). A total of 15 subjects in chitosan group and 6 subjects in placebo group had hypertension, diabetes mellitus, dyslipidemia or their combination.

**Table 1:** Demographics of study participants

Subject characteristic	Chitosan (*N* = 64)	Placebo (*N* = 32)
Age (years) [range]	35.53 ± 11.23 [19–63]	36.28 ± 10.49 [18–56]
Gender (male /female)	M = 25; F = 39	M = 11; F = 21
Weight (Kg) [range]	80.13 ± 11.47 [54.0–106.5]	80.54 ± 12.68 [59.8–116.0]
Height (M) [range]	1.61 ± 0.10 [1.38–1.82]	1.61 ± 0.09 [1.47–1.83]

Values are expressed as Mean ± Standard deviation (SD). Gender expressed as absolute number. *N* = number of patients. Data are presented as descriptive statistics

### Efficacy analyses

Reduction in the mean body weight over a period of 45 and 90 days intervention for chitosan and placebo group was assessed. In chitosan group, body weight was reduced from 80.13 ± 11.47 kg at baseline to 77.75 ± 11.56 at day 45 and 76.89 ± 11.88 kg at the end of 90 days, which was statistically significant (p < 0.0001) when compared with baseline measurements. While in placebo group the body weight was 80.54 ± 12.68 kg at baseline, which minimally changed to 80.89 ± 12.15 kg at 45 days and 80.76 ± 12.31 kg at the end of 90 days of treatment, which was statistically non-significant. It is noteworthy that in chitosan group, the percentage of subjects who reduced body weight in the range of up to 2 kg, 2–4 kg and >4 kg was 54.2 % (*n* = 32), 28.8 % (*n* = 17) and 10.2 % (*n* = 6) at the end of day 45 and 10.7 % (*n* = 6), 48.2 % (*n* = 27) and 33.9 % (*n* = 19) at the end of day 90, respectively. While in the placebo group, the percentage of subjects who reduced body weight in the same range was 41.9 % (*n* = 13), 12.9 % (*n* = 4) and 0 % (*n* = 0) at day 45 and 40.0 % (*n* = 12), 10.0 % (*n* = 3) and 3.3 % (*n* = 1) at the end of day 90, respectively. Only about 6.8 % (*n* = 4) subjects at day 45 and 7.1 % (*n* = 4) subjects at day 90 were non-responders in chitosan group, while in placebo group, the percentage of non-responders were 45.2 % (*n* = 14) and 46.7 % (*n* = 14) subjects at day 45 and day 90, respectively.

**Fig. 2:**
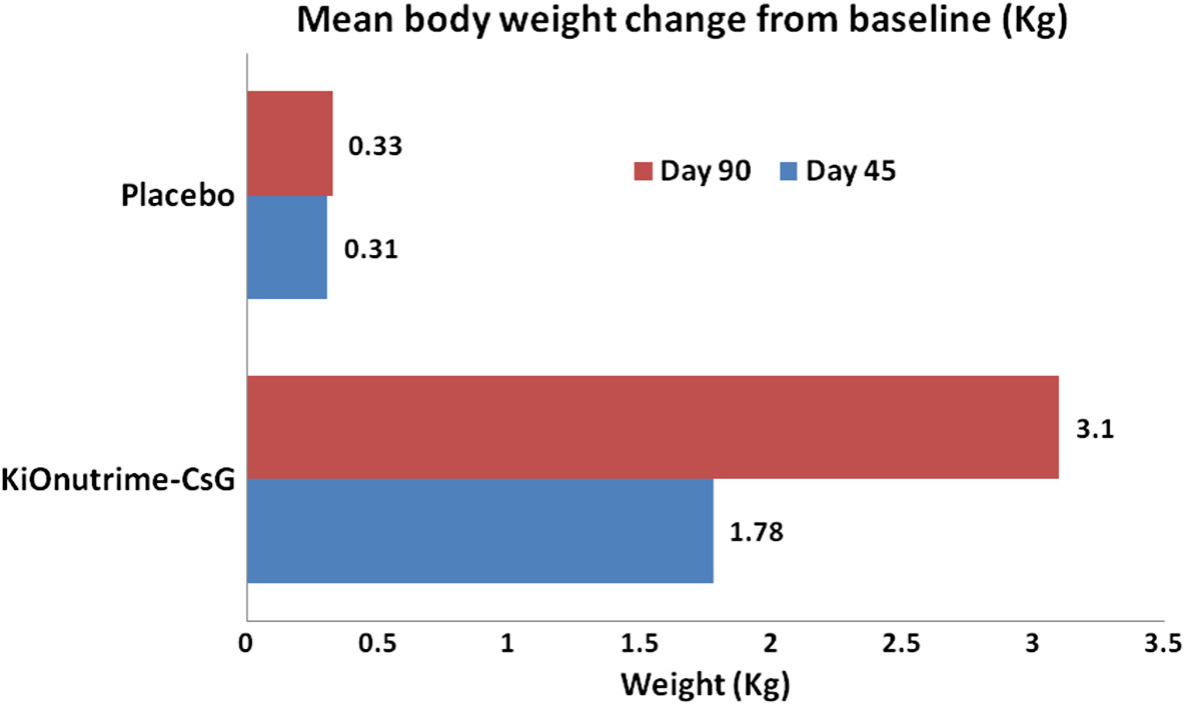
Mean body weight changes from baseline

In chitosan group, the mean change in body weight was -1.78 ± 1.37 kg (range: -5.30 to 0.80 kg) and -3.10 ± 1.95 kg (range: -9.00 to 1.90 kg) at day 45 and day 90, respectively. These results were significantly different (*p* < 0.0001) as compared to placebo where mean change in body weight was -0.31 ± 1.30 kg (range: -3.00 to 2.50 kg) and -0.33 ± 1.51 kg (range: -4.60 to 2.80 kg), respectively (Fig. 2).Table 2 shows the comparison between body weights in both the groups.

**Table 2:** Effect of treatments on body weight at day 45 and day 90 (in Kg)

Visit	Chitosan (*N* = 64)	Placebo (*N* = 32)	*P* value between group
Day 0 (n = 64, 32)	80.13 ± 11.47	80.54 ± 12.68	0.87
Day 45 (n = 59, 31)	77.75 ± 11.56*	80.89 ± 12.15	0.43
Day 90 (n = 56, 30)	76.89 ± 11.88*	80.76 ± 12.31	0.35
Change from baseline			
Day 45 (n = 59,31)	−1.78 ± 1.37@	−0.31 ± 1.30	< 0.0001
Day 90 (n = 56, 30)	−3.10 ± 1.95@	−0.33 ± 1.51	< 0.0001

Values are expressed as Mean ± Standard deviation (SD); *p < 0.0001 as compared to baseline (Within group comparison); @ - statistically significant as compared to placebo at day 45 and day 90; *N* = Number of subjects in each treatment group. *n* = number of subjects with non-missing values at respective visit

### Other study parameters

Table 3 describes the absolute values of each study parameters at baseline, at day 45 and day 90 and Table 4 describes the change in each parameter at day 45 and day 90 from baseline.

**Table 3:** Study parameters values at baseline, day 45 and day 90 in treatment groups

Study parameters	Chitosan (mean ± SD) [range]			Placebo (mean ± SD) [range]		
	Day 0 (*N* = 64)	Day 45 (*N* = 59)	Day 90 (*N* = 56)	Day 0 (*N* = 32)	Day 45 (*N* = 31)	Day 90 (*N* = 30)
Body mass Index (Kg/m^2^)	30.93 ± 2.69 [26.49 – 35.19]	30.20 ± 2.90* [25.32 – 35.08]	29.71 ± 3.07* [24.88 – 35.64]	30.91 ± 2.72 [26.23 – 34.95]	30.95 ± 2.62 [26.29 – 34.95]	30.83 ± 2.64 [26.36 – 35.09]
Body fat (%)	37.88 ± 6.86 [25.20 – 49.50]	37.36 ± 7.03* [25.00 – 50.30]	36.65 ± 7.25* [24.30 – 49.40]	37.96 ± 7.83 [23.70 – 49.30]	38.02 ± 8.00 [23.70 – 49.30]	38.07 ± 8.09 [23.50 – 49.10]
Visceral fat (%)	10.80 ± 3.52 [4 – 20]	10.47 ± 3.3* [4 – 20]	9.71 ± 3.33* [4 – 20]	10.53 ± 2.97 [4 – 17]	10.55 ± 2.75 [5 – 17]	10.43 ± 2.87 [5 – 17]
Muscle mass (Kg)	47.52 ± 9.62 [32.20 – 68.30]	46.74 ± 9.50* [32.00 – 64.50]	46.84 ± 9.57* [31.70 – 64.20]	46.95 ± 10.79 [34.70 – 81.10]	47.04 ± 11.17 [35.20 – 83.80]	47.50 ± 11.01 [35.20 – 83.00]
Upper abdominal circumference (cm)	97.70 ± 7.96 [80.00 – 114.0]	96.61 ± 7.99* [80.00 – 113.0]	95.68 ± 8.40* [77.00 – 112.0]	95.75 ± 9.22 [73.00 – 117.0]	95.55 ± 9.13 [74.00 – 115.0]	95.73 ± 9.09 [74.00 – 115.0]
Hip circumference (cm)	110.35 ± 9.30 [89.00 – 130.0]	109.2 ± 9.77* [88.00 – 128.0]	108.3 ± 9.96* [88.00 – 128.0]	108.59 ± 14.80 [48.00 – 138.0]	109.0 ± 13.91 [50.00 – 138.0]	108.3 ± 13.79 [50.00 – 137.0]
Waist circumference (cm)	102.80 ± 7.70 [84.00 – 118.0]	101.7 ± 7.69* [85.00 – 117.0]	100.5 ± 8.05* [82.00 – 115.0]	102.72 ± 10.14 [67.00 – 124.0]	102.2 ± 10.27 [68.00 – 124.0]	102.3 ± 10.12 [68.00 – 124.0]
Waist to hip ratio	0.93 ± 0.07 [0.80 - 0.95]	0.93 ± 0.07 [0.80 – 0.97]	0.92 ± 0.07 [0.79 – 0.96]	0.96 ± 0.12 [0.75 – 0.98]	0.94 ± 0.10 [0.74 – 0.98]	0.95 ± 0.10 [0.82 – 0.98]

Values are expressed as Mean ± Standard deviation (SD). *p < 0.0001 as compared to baseline (Within group comparison). *N* = Number of patient in each treatment group

**Table 4:** Mean change from baseline in study parameters

	Visit	Chitosan (mean ± SD) [range]	Placebo (mean ± SD) [range]	*P* value between groups
Body mass index(Kg/m^2^)	Day 45 (*n* = 59, 31)	-0.69 ± 0.53^@^ [-2.15 – 0.32]	-0.11 ± 0.50 [-1.19 – 1.01]	< 0.0001
	Day 90 (*n* = 56, 30)	-1.20 ± 0.76^@ ^[-3.65 – 0.73]	-0.11 ± 0.59 [-1.81 – 1.14]	< 0.0001
Body fat (%)	Day 45 (*n* = 59, 31)	-0.38 ± 1.17^@ ^[-6.60 – 2.80]	-0.11 ± 1.12 [-3.70 – 2.80]	0.034
	Day 90 (*n* = 56, 30)	-0.98 ± 1.27^@^ [-6.80 – 2.60]	-0.05 ± 0.98 [-2.70 – 2.30]	< 0.0001
Visceral fat (%)	Day 45 (*n* = 59, 31)	-0.38 ± 0.71 [-3.0 – 1.0]	-0.19 ± 0.54 [-2.0 – 1.0]	0.127
	Day 90 (*n* = 56, 30)	-1.28 ± 1.12^@^ [-4.0 – 1.0]	-0.43 ± 0.85 [-2.0 – 2.0]	0.001
Muscle mass (Kg)	Day 45 (*n* = 59, 31)	-0.48 ± 1.51 [-11.10 – 1.20]	-0.20 ± 0.89 [-2.50 – 2.70]	0.253
	Day 90 (*n* = 56, 30)	−0.74 ± 1.57^@^ [−11.50 – 1.20]	-0.08 ± 1.16 [-3.20 – 3.10]	0.0008
Upper abdominal circumference (cm)	Day 45 (*n* = 59, 31)	-0.92 ± 1.25^@^ [-6.0 – 2.0]	-0.35 ± 0.95 [-3.0 – 1.0]	0.020
	Day 90 (*n* = 56, 30)	-2.17 ± 1.98^@^ [-8.0 – 2.0]	-0.50 ± 1.10 [-3.0 – 1.0]	< 0.0001
Hip circumference (cm)	Day 45 (*n* = 59, 31)	-1.05 ± 1.05^@^ [-4.0 – 0.0]	-0.41 ± 1.14 [-4.0 – 2.0]	0.0024
	Day 90 (*n* = 57, 30)	-2.07 ± 1.51^@^ [-6.0 – 2.0]	-0.66 ± 1.24 [-3.0 – 2.0]	< 0.0001
Waist circumference (cm)	Day 45 (*n* = 59, 31)	-0.91 ± 1.68^@^ [-8.00 – 6.00]	-0.64 ± 1.22 [-1.00 – 6.00]	0.027
	Day 90 (*n* = 57, 30)	-1.97 ± 2.20^@^ [-8.00 – 7.00]	-0.90 ± 1.47 [-1.00 – 7.00]	< 0.0001
Waist to hip ratio	Day 45 (*n* = 59, 31)	-0.0027 ± 0.02 [-0.13 – 0.04]	-0.0035 ± 0.01 [-0.03 – 0.03]	0.091
	Day 90 (*n* = 57, 30)	-0.0010 ± 0.02 [-0.13 – 0.07]	-0.0033 ± 0.01 [-0.02 – 0.04]	0.768

Values are expressed as Mean ± Standard deviation (SD); *N* = Number of subjects in each treatment group; @ - statistically significant when compared to placebo; *n* = number of patients with non-missing values at respective visit

Mean BMI decreased significantly (*p* < 0.0001) over the period of 90 days in chitosan group (30.93 ± 2.69 at baseline to 30.20 ± 2.90 at day 45 and 29.71 ± 3.07 at day 90), while in placebo group it was decreased minimally but was not significant (*p* = 0.3846) as compared to baseline values (30.91 ± 2.72 at baseline to 30.95 ± 2.62 at day 45 and 30.83 ± 2.64 at day 90) (Table 3). Mean change in the reduction of BMI from baseline was significantly higher in chitosan group on day 45 and day 90 as compared to subjects receiving placebo (Table 4).In chitosan group the mean changes in BMI at day 45 were found to be in the range of -2.15 to +0.32 (mean -0.69), while the same for placebo was in a range of -1.19 to +1.01 (mean -0.11). After 90 days of administration, there was a further reduction in BMI in chitosan group which was in the range of -3.65 to +0.73 (mean -1.20) as compared to -1.81 to +1.14 (mean -0.11) in placebo group.

Similarly, body fat was significantly reduced (*p* < 0.0001) in subjects administered with chitosan at the end of 90 days (37.88 ± 6.86, 37.36 ± 7.03 and 36.65 ± 7.25 at baseline, day 45 and day 90 respectively) (Table 3), while it was increased slightly in placebo group (*p* = 0.5684). However, there was no statistical difference between both treatments at any time points. Mean changes in body fat reduction from baseline in chitosan group as compared to placebo group at day 45 (-0.38 ± 1.17 % vs-0.11 ± 1.12 %) and day 90 (-0.98 ± 1.27 % vs-0.05 ± 0.98 %) were significantly different (Table 4). This mean change in body fat reduction was in the range of -6.60 to +2.80 % and -6.80 to +2.60 % in chitosan group, while in placebo group it was -3.70 to +2.80 % and -2.70 to +2.30 % at day 45 and day 90, respectively.

Visceral fat significantly decreased (*p* < 0.0001) in subjects administered with chitosan at day 45 (10.47 ± 3.38 %) from baseline (10.80 ± 3.52). This further decreased to 9.71 ± 3.33 % at the end of 90 days administration (Table 3). In placebo group, however, visceral fat remained unchanged at day 45 (10.55 ± 2.75 %) and at the end of 90 days (10.43 ± 2.87 %). Again, when compared between treatments, the values were statistically non-significant. But when mean changes in reduction of visceral fat from baseline was compared (Table 4), it was observed that chitosan showed significantly higher (*p* < 0.001) reduction in visceral fat as compared to placebo on day 90 (1.28 ± 1.12 % vs 0.43 ± 0.85).

We found that muscle mass decreased in chitosan group (47.52 ± 9.62, 46.74 ± 9.50 and 46.84 ± 9.57 at baseline, day 45 and day 90 respectively) and increased in placebo group over a 90-days administration (46.95 ± 10.79, 47.04 ± 11.17 and 47.50 ± 11.01 at baseline, day 45 and day 90 respectively) (Table 3). However, there was no significant difference between treatments (*p* = 0.581, 0.798, 0.969 at day 0, 45 and 90 respectively). But when mean changes in muscle mass from baseline was compared, it was found that at day 90 there was significant difference (*p* = 0.0008) between the groups (-0.74 ± 1.57 vs. -0.08 ± 1.16). This can also be observed by reduction of muscle mass in the range of -11.50 to +1.20 in chitosan group as compared to -3.20 to +3.10 in placebo group at day 90 (Table 4).

The reduction in body weight caused a comparable decrease in anthropometric measurement as well. There was significant mean reduction in upper abdominal circumference, hip circumference and waist circumference at day 45 (*p* < 0.0001) and day 90 (*p* < 0.0001) from baseline in subjects treated with chitosan capsules (Table 3). On the contrary, there was no statistical significant reduction in upper abdominal circumference, hip circumference and waist circumference in patients treated with placebo on day 45 and day 90. Mean change in reduction from baseline in upper abdominal circumference (-0.92 ± 1.25 and -2.17 ± 1.98 vs. -0.35 ± 0.95 and -0.50 ± 1.10), hip circumference (-1.05 ± 1.05 and -2.07 ± 1.51 vs. -0.41 ± 1.14 and -0.66 ± 1.24) and waist circumference (-0.91 ± 1.68 and -1.97 ± 2.20 vs. -0.64 ± 1.22 and -0.90 ± 1.47) was significantly (*p* < 0.0001) greater in subjects treated with chitosan than with placebo at day 45 and day 90, respectively (Table 4). There was no significant change in waist to hip ratio in both treatment groups at day 45 and day 90 (-0.0027 ± 0.02 and -0.0010 ± 0.02 vs. -0.0035 ± 0.01 vs.-0.0033 ± 0.01 at p = 0.091 and 0.768).

HbA1c level at baseline was compared with post-administration measurements at day 45 and day 90 to assess the efficacy of chitosancapsules. In this study, HbA1c level was significantly decreased at day 45 (5.72 ± 0.78 %) and day 90 (5.74 ± 0.83 %) in chitosan group as compared to its baseline value (5.89 ± 0.83), which was statistically significant (*p* = 0.0327). However, within placebo group there was statistically significant reduction (*p* = 0.0334) observed at day 45 only as compared to its baseline values, while at day 90 it again increased and was statistically non-significant (*p* = 0.8269) as compared to its baseline values (Table 5). Further analysis revealed that, there were 17 subjects whose HbA1c levels were above 6 % (mean: 6.55 %; range: 6 to 8.2 %) while the remaining subjects had HbA1c levels below 6 % (mean: 5.47 %; range: 4.3 to 5.9 %) at baseline. After 90 day treatment with chitosan, HbA1c level significantly decreased in those17 subjects (mean: 6.04 %; range: 5.1 to 6.8 %) while in the remaining subjects, it was unchanged throughout the study period (mean: 5.48 %; range: 4.7 to 5.9 %). This shows that chitosan was effective in reducing HbA1c levels in subjects who were having higher glycaemic value initially, while subjects with normal glycaemic levels were unaffected.

**Table 5:** Comparison in lipid profile (TG, HDL, LDL and VLDL) and HbA1C levels

Visit	Chitosan (*N* = 56)	Placebo (*N* = 30)	*P* value between groups
TG (mg/dl)			
Day 0	145.2 ± 64.35	153.1 ± 76.44	0.753
Day 45	143.7 ± 76.33	160.5 ± 92.92	0.494
Day 90	138.8 ± 63.81	170.3 ± 102.8	0.203
HDL(mg/dl)			
Day 0	41.47 ± 7.50	42.87 ± 10.28	0.448
Day 45	44.38 ± 12.01	41.14 ± 9.55	0.285
Day 90	43.17 ± 11.65	42.08 ± 10.74	0.944
LDL (mg/dl)			
Day 0	106.9 ± 27.80	107.2 ± 30.39	0.959
Day 45	114.9 ± 42.44* (*p* = 0.0213)	113.7 ± 38.66	0.746
Day 90	109.9 ± 31.64	115.6 ± 31.93* (*p* = 0.0266)	0.428
VLDL (mg/dl)			
Day 0	29.04 ± 12.87	30.62 ± 15.29	0.746
Day 45	28.74 ± 15.27	32.10 ± 18.59	0.494
Day 90	27.76 ± 12.76	34.07 ± 20.55	0.203
HbA1C (%)			
Day 0	5.89 ± 0.83	5.89 ± 0.75	0.912
Day 45	5.72 ± 0.78* (*p* = 0.0225)	5.80 ± 0.68* (*p* = 0.0419)	0.661
Day 90	5.74 ± 0.83* (*p* = 0.0343)	5.88 ± 0.57	0.138

Values are expressed as Mean ± Standard deviation (SD). * = as compared to baseline (Within group comparison). *N* = Number of patient in each treatment group

Analysis of daily food intake for the period of 15 days (day 1–5, day 41–45 and day 86–90) for calorie intake showed there was no significant change, in either group, during this study. The mean caloric intake in chitosan group for day 1–5 was 1784 kcal, for day 41–45 was 1797 kcal and for day 86–90 was 1750 kcal. While the same for placebo group was 1761 kcal, 1701 kcal and 1677 kcal, respectively.

### Safety analyses

Lipid levels in both treatment groups are described in Table 5. Although LDL levels increased in chitosan group at day 45 and in placebo group at day 90, in general the results were clinically non-significant as this increase in LDL can be attributed to only two of the subjects; one in chitosan group and one in placebo group who showed transient increase in their LDL levels.

The SF-36 analysis shows that the mean PCS score and mean MCS score obtained in chitosan group at day 0 were 40.99 ± 6.51 and 48.34 ± 6.77, respectively and at day 90 were 51.32 ± 7.23 and 49.10 ± 7.08, respectively. The mean PCS score and mean MCS score obtained in placebo group at day 0 were 41.26 ± 5.78 and 46.16 ± 7.77, respectively and at day 90 were 43.19 ± 7.50 and 47.45 ± 6.60, respectively.Assessment of Quality of Life (QoL) using SF-36 questionnaire showed statistical significant (*p* < 0.0001) increase in QoL score in subjects from chitosan group as compared to the placebo group from baseline to day 90, which depicts improvement in the QoL (Table 6).

**Table 6:** Effect of treatment groups on quality of life score

Visit	Chitosan (mean ± SD) [range]	Placebo (mean ± SD) [range]	*P* value
PCS Score			
Day 0 (*n* = 58, 31)	40.99 ± 6.51 [22.80 − 56.90]	41.26 ± 5.78 [33.10 − 57.70]	0.869
Day 90 (*n* = 59, 31)	51.32 ± 7.23*^@^ [32.10 − 61.80]	43.19 ± 7.50[31.20 − 56.40]	< 0.0001
Change from baseline (PCS Score)			
Day 90 (*n* = 59, 31)	10.58 ± 8.07^@^ [-27.70 – 8.50]	1.93 ± 5.69 [−17.60 – 6.30]	< 0.0001
MCS score			
Day 0 (*n* = 58, 31)	48.34 ± 6.77 [27.20 − 60.50]	46.16 ± 7.77 [17.70 − 61.90]	0.173
Day 90 (*n* = 59, 31)	49.10 ± 7.08 [33.20 − 61.60]	47.45 ± 6.60 [27.90 − 62.40]	0.283
Change from baseline (MCS Score)			
Day 90 (*n* = 59, 31)	0.98 ± 6.73 [-20.80 − 16.70]	1.28 ± 8.81 [-34.30 − 10.60]	0.4755

Values are expressed as Mean ± Standard deviation (SD). * = statistically significant as compared to baseline. @ - statistically significant as compared to placebo at day 90. *N* = Number of subjects in each treatment group

There were a total of 10 adverse events (AEs) recorded during the study period: four in placebo group and six in chitosan group. In chitosan group reported AEs were common cold, hypertriglyceridemia, body ache, constipation (2 subjects) and hypertension, while in placebo group, the reported AEs were mild headache (2 subjects), hypertriglyceridemia and fracture. All adverse events were mild in nature and unrelated to the study treatment.There was no statistically significant difference in laboratory parameters (SGOT, SGPT, serum creatinine and urea) from baseline to day 90 in both chitosan and placebo groups. No dropout was observed due to AEs, which states that overall the study treatment was safe and well tolerated by all study subjects.

## Discussion

This study demonstrates that administration of chitosan (KiOnutrime-CsG® capsules, 500 mg, 5 capsules/day in three divided doses) results in a significant mean weight loss of about 3 kg without diet restriction over a period of 90 days. The observed weight loss in chitosan group is in contrast to only 0.3 kg weight loss in placebo group. Also significant was the percentage of subjects who lost between 5 and 10 % of body weight after 90 days compared to placebo group (32.4 and 3.3 %, respectively). Although some studies demonstrated that reduction in body weight by administration of chitosan can be achieved in individuals given a hypocaloric or standardized diet [14, 29], other studies show efficacy of chitosan for persons without diet restrictions [10, 23, 30, 31]. The results of our study confirm that indeed significant weight loss can be achieved in subjects adhering to a non-restrictive diet [10, 23, 30, 31]. Reasons for the difference in results in our study with other reported studies could be difference in diets, dosage and timing of chitosan administration or protocol variability such as life style recommendations.

One factor which is important to consider is the timing of chitosan ingestion before meals. It is typically recommended that chitosan supplements be ingested approximately 15 min to 1 h prior to a meal in order to allow sufficient time for chitosan to dissolve in the stomach acid[18]. In our study, the dosage was one capsule 15 min before breakfast and two capsules each 15 min before lunch and dinner. This allowed sufficient time for it to dissolve properly and efficiently bind the fats present in the meal, which resulted in observed weight loss.

Body weight gain and increase in BMI are the key clinical features of obesity. BMI correlates fairly well with total body fat on a population basis [32]. The overweight (BMI 25.0 to 29.9 kg/m^2^) and obese (BMI ≥30 kg/m^2^) individuals have higher body fat together with increased risk of cardiovascular and other metabolic disorders. In this study we found that after 90 day administration with chitosan, there was 10.91 fold reduction in BMI compared to placebo group. The implications of this result are that the subjects, who were initially classified as obese, can now be defined as overweight as their mean BMI fell below 30 kg/m^2^.

It is well known that weight reduction in subjects with obesity has a marked effect on the regulation of lipolysis [33] and weight loss shows good correlations with several of the circumferences [34] that were measured in present study. Also, in one of the gastric bypass study conducted by Sjostrom and colleagues [35], it was found that the profound weight loss experienced by the subjects resulted from a global decrease in body fat rather than localised loss. Also, hypocholesterolemic properties of chitosan decrease the risk of atherosclerosis and other cardiovascular dysfunctions [36]. Chitosan, by the virtue of its property to bind fat and triglycerides, may also have caused the disturbances in regulation of lipolysis resulting in lowering of body fat and visceral fat observed in our study.

Reduction of muscle mass by chitosan was observed in this study which is reduced in an average of 0.74 kg over a period of 90 days. Although there is a statistically significant reduction, this has not produced any clinically relevant adverse effects over a period of 90 days.

It is already reported that chitosan can regulate lipids with benefit on anthropometric parameters [37]. Also, in one of the study conducted over a period of five years, it was confirmed that weight gain and weight loss are associated with changes in the anthropometric measurements and waist to hip ratio (WHR) in both genders [38]. The reduction in body composition and anthropometric parameters observed in our study can be attributed to general reduction in body weightpossibly due to reduction in fat absorption [39] by chitosan.

Practically no significant change was observed in serum triglyceride, LDL and VLDL throughout the test period while HDL was slightly increased in chitosan group (non-significant). It is well known that Low-density lipoproteins (LDL) are considered as important risk factors for cardiovascular diseases (CVD), while highdensity lipoproteins (HDL) are well recognized for their putative role in reverse cholesterol transport [40]. Since HDL-cholesterol is more metabolisable into bile acid than LDL-cholesterol [41], it is presumed that a deficiency of bile acid in the body due to binding with chitosan would accelerate the conversion of cholesterol to bile acid, which may result in an increase of HDL-cholesterol. In a similar previous study where effects of chitosan was studied on lipids and lipoproteins, it was found that chitosan increased HDL level up to 14 % during the 4-month study period [42].

Obesity is a multi-factorial disorder, which is often associated with many other significant diseases such as diabetes, inflammation, hypertension and other cardiovascular diseases; there is a consistent graded relationship between increased BMI and prevalence of non-insulin dependent diabetes mellitus (NIDDM) and insulin resistance [43]. It is established that inflammation, diabetes and obesity are interrelated and a person with diabetes are predisposed to obesity and metabolic syndrome. HbA1C reflects the long-term glycaemic level and is a marker for progression of diabetes. In the present study, we observed that chitosan was able to lower the HbA1C level to less than 6 % during the 90-days study period. It has been reported that chitosan significantly reduced postprandial blood glucose levels in both animal and in vitro models [44] as well as in humans [45]. This may the reason for the observed decrease in HbA1c levels in our study. Interestingly, this reduction was mainly observed in subjects who were initially having high HbA1C levels, while subjects with normal HbA1C levels at baseline were unaffected by chitosan. However, more clinical studies are required to confirm this effect of chitosan in large diabetic population.

The results of SF-36 QoL score showed that there was significant improvement in mean PCS score in chitosan group which reflects improvement in physical morbidity and adaptation to obesity. However, mean MCS score failed to improve with the treatment. This may be due to failure to evaluate the impact that excess weight would have on obesity-specific aspects of QoL score during the baseline evaluations [46]. This might explain why no effect of decrease in BMI was detected on MCS despite it being recognised that people who are overweight or obese are more likely to suffer from discrimination and depression [47]. Another possible explanation may be that people who are very overweight and obese may need to loose in excess of 10 % of their body weight in order to experience a positive impact on QoL [48]. However, only one of the subjects in our study showed weight loss of more than 10 %, thus explaining the differences in PCS and MCS score.

## Conclusion

In summary, we conclude that KiOnutrime-CsG® capsule, containing 500 mg of chitosan from fungal origin, was able to reduce the mean body weight up to 3 kg during the 90-days study period. Together with this, there was also improvement in body composition, anthropometric parameters and HbA1C, reflecting overall benefits for the overweight individuals. Additionally, there was also improvement in QoL score. KiOnutrime-CsG® capsulewas also found to be safe and well tolerated by all study participants.

## Availability of supporting data

The data set(s) supporting the results of this article is (are) included within the article.

## Abbreviations

BMI: body mass index
HbA1C: glycated haemoglobin
IASO: International Association for the Study of Obesity
IOTF: International Obesity Task Force
QoL: quality of life
WHR: waist to hip ration
